# Floes, the marginal ice zone and coupled wave-sea-ice feedbacks

**DOI:** 10.1098/rsta.2021.0252

**Published:** 2022-10-31

**Authors:** Christopher Horvat

**Affiliations:** ^1^ The University of Auckland, Auckland, New Zealand; ^2^ Institute at Brown for Environment and Society, Providence, RI, USA

**Keywords:** sea ice, ocean surface waves, polar climate

## Abstract

Marginal ice zones (MIZs) are qualitatively distinct sea-ice-covered areas that play a critical role in the interaction between the polar oceans and the broader Earth system. MIZ regions have high spatial and temporal variability in oceanic, atmospheric and ecological conditions. The salient qualitative feature of MIZs is their composition as a mosaic of individual floes that range in horizontal extent from centimetres to tens of kilometres. Thus the floe size distribution (FSD) can be used to quantitatively identify and describe them. Here, the history of FSD observations and theory, and the processes (particularly the impact of ocean waves) that determine floe sizes and size distribution, are reviewed. Coupled wave-FSD feedbacks are explored using a stochastic model for thermodynamic wave-sea-ice interactions in the MIZ, and some of the key open questions in this rapidly growing field are discussed.

This article is part of the theme issue ‘Theory, modelling and observations of marginal ice zone dynamics: multidisciplinary perspectives and outlooks’.

## 1. The marginal ice zone

Marginal ice zones (MIZs) are enigmatic, variable and heterogeneous regions covered by sea ice that ‘separate open water and pack ice’ [1] but that elude simple quantitative description. Typically, the MIZ is contrasted with the region of high-concentration ‘pack’ sea ice observable from passive microwave satellites, that covers the Arctic Ocean basin and hugs the coast of the Antarctic continent [2–6]. MIZs, however, are dynamically distinct areas. *In situ*, they are readily characterized by two key observable features: waves and floes [7–9].

The first^1^ joint observation of waves, floe diameter and sea ice concentration [7] declared: ‘analysis indicates that [sea ice concentration] is not an important parameter, [thus] errors in estimating the ice cover will not affect the conclusions’. While the compactness of sea ice *is* clearly relevant to the dynamical interactions between sea ice and the rest of the coupled system, in hindsight, it is also now clear that sea ice concentration alone is not sufficient to describe MIZ dynamics, either qualitatively or quantitatively.

The influence of ocean surface waves is also used to define the MIZ [10]. Waves can be observed propagating long distances within the sea ice [8,9,11–15]. A companion review in this special issue discusses the theory and observation of waves in the MIZ [16]. Recently, new satellite platforms have allowed the observation of waves in sea ice at high resolution and at global scales, making it feasible to quantify global MIZ extent [13,15,17]. Yet as sea ice is an efficient damper of high-frequency wave energy, waves in sea ice are often long-period swell waves, episodically generated by storms or mesoscale weather events [12,18–20]. At any instant, the area of ice-covered regions with energetic ocean surface waves can be much smaller than that ‘influenced’ by waves more generally. In global measurements with the ICESat-2 altimeter, the MIZ was defined as those regions where waves were identified at least 7.5% of the time [6]—and of 4402 individual observations of sea ice heights in the Southern Ocean, just 304 (7.0%) were identified visually as having waves in them [15]. The MIZ is dynamic, and ICESat-2 overflights inconsistently sample the constantly varying sea ice surface. Thus we must also seek a consistent, more slowly evolving feature that sets the MIZ apart from other sea-ice-covered areas.

**Figure 1.**
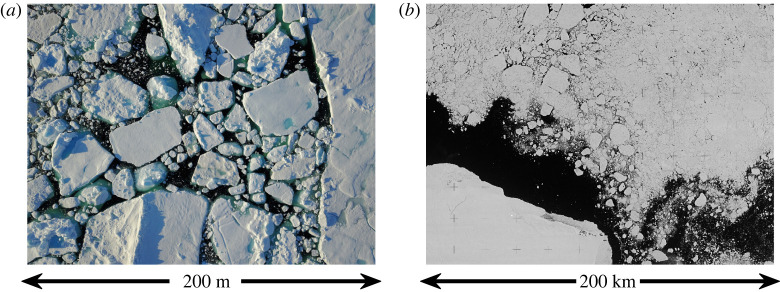
Floes in the MIZ. (*a*) Composite of small floes observed during the *Enduring Ice* expedition to Nares Strait. Floes range in scale from sub-metre to hundreds of metres. Horizontal scale is approximately 200 m at approximately 5 cm resolution.(*b*) Declassified Keyhole-7 imagery of floes surrounding Zemlya Georga in the Arctic Ocean. Floes range in scale from metres to kilometres. Horizontal scale is approximately 200 km at approximately 10 m resolution. (Online version in colour.)

One such obvious feature is floes. Sea ice in the MIZ is comprised of a myriad of individual floating pieces with unique sizes and shapes (figure 1). Sea ice is, at all scales, a floe composite, but in the MIZ these floes span a scale range from centimetres to kilometres and directly influence sea ice evolution and ice-ocean-atmospheric coupling. Fields of energetic waves bend sea ice, fracturing it into smaller pieces and leaving a field of small floes that alters sea ice properties as well as the transmission and scattering of wave energy [9,21–24]. These smaller floes are then more prone to melting [25]. Waves also influence the formation of sea ice, [26,27], herding frazil crystals into size-limited pancake floes. Thus areas ‘under the influence’ of waves bear the imprint of wave events in the form of fields of fractured or small sea ice floes, an imprint apparent in satellite imagery (figure 1*b*).

To quantify geometric variability in the MIZ, floes are described in terms of their floe size distribution (FSD). The FSD is a probability distribution that quantifies the statistics of horizontal scales in the floe mosaic. Building from an legacy of ship, helicopter, and satellite-based observations, current inquiry into the FSD spans a range of observational, theoretical and modelling approaches—and the study of the FSD and its impact on polar climate systems are key questions facing climate modellers. Below, the concept and definitions of the FSD are summarized in §2, with past and present observational efforts to describe it in §3. Recent approaches to FSD modelling, particularly its interaction with ocean waves, are highlighted in §4. FSD-related feedbacks and their impact on other aspects of the coupled system are then explored in §5.

## 2. The floe size distribution

The evolution of floe sizes in a geographical region A can be characterized by an area-weighted FSD [28]. The FSD is a function, n(r) dr, equal to the fraction of A comprised of sea ice with a characteristic horizontal length scale between r and r+dr. A floe’s ‘size’ can be determined from imagery. Often this is using its ‘mean caliper diameter’, the average width a set of calipers would have to be to grasp the floe [28–31]. For simplicity, floes are represented here as perfect circles with an area $A=\pi r^{2}$.

The FSD obeys a conservation equation,

2.1 $$c=\int_{0}^{\infty }n(r)\;dr,$$

where c is the sea ice concentration over A. The first moment of n(r) is the ‘representative radius’,

2.2 $$\bar{r}=\int_{0}^{\infty }rn(r)\;dr.$$

While $\bar{r}$ is not the (population) mean floe size, which is obtained from the number distribution of floes (see below), it is used in several parameterizations of FSD processes (see §4).

It is common also to describe an ‘FSD’ as the *number density* N(r) of floes. Then N(r) is equal to the total number of floes per square metre of A with size between r and r+dr. If floes are all perfect circles with radius r,

2.3 $$N(r)=\frac{n(r)}{\pi r^{2}}.$$

The floe number density may be conserved in some physical situations that the FSD is not, like when sea ice melts laterally or advects. On the other hand, floe numbers can change when total floe area does not, as in the case of sea ice fracture. Both n(r) and N(r) are connected to the perimeter of sea ice floes per square metre, P (units 1 m${\;}^{-1}$),

2.4 $$P=\int_{0}^{\infty }\frac{2n(r)}{r}\;dr=\int_{0}^{\infty }2\pi rN(r)\;dr,$$

which has units 1 m${\;}^{-1}$. P is important for determining lateral melt rates in models [25,32–34].

Another common way of visualizing the FSD [30] is the complementary cumulative number distribution C(r),

2.5 $$C(r)=\int_{0}^{r}N(r)\;dr=\int_{0}^{r}\frac{n(r)}{\pi r^{2}}\;dr.$$

The utility of C(r) is in analogy to fractal systems which typically obey a power-law scaling. The hypothesis that the FSD follows a power law has long been applied to data, though it is contentious. Prescriptive methods for analysing multi-scale FSD measurements are reviewed in [31], and also discussed in [35].

### (a) The floe size and thickness distribution

Each floe has both a horizontal and vertical size. Thus to fully characterize the sub-gridscale distribution of floes requires defining the floe size and thickness distribution [32, FSTD], f(r,h). The distribution f(r,h) dr dh is the fraction of A that is comprised of sea-ice floes with thickness between h and h+dh and size between r and r+dr. It is related to the ice thickness distribution, g, and n,

$$g(h)=\int_{0}^{\infty }f(r,h)\;dr\;\mathrm{and}\;n(r)=\int_{0}^{\infty }f(r,h)\;dh.$$

It can therefore be used to obtain both sea ice concentration, c, and ice volume per unit area, V, over A as,

2.6 $$c=\int_{0}^{\infty }\int_{0}^{\infty }f(r,h)\;dr\;dh\;\mathrm{and}\;V=\int_{0}^{\infty }\int_{0}^{\infty }hf(r,h)\;dr\;dh.$$

## 3. Observing the floe size distribution

Observations of floe sizes have long been recorded during sea-going voyages. A typical procedure is one described by Robin [7]. Ice lookouts record the average diameter of the pieces of sea ice encountered by an ship, usually estimated by eye and collated over some area. The WMO [10] designs a protocol for such measurements taken from a ship, with a series of sea-ice-floe size/types. Because of the subjective quality of these measurements and their reliance on trained observers, such measurements are challenging to adopt into scientific studies of the FSD beyond describing local conditions for supplementing more detailed measurements.

### (a) From imagery

Modern observations of the FSD use a wide range of satellite and aerial platforms. Some have used ship-or-buoy-borne cameras (e.g. [27]), some have used helicopters (e.g. [36], who also used ship-based cameras), and many more have used satellite imagery or SAR data (reviews on the subject in [31,37] contain a list of many such references).

**Figure 2.**
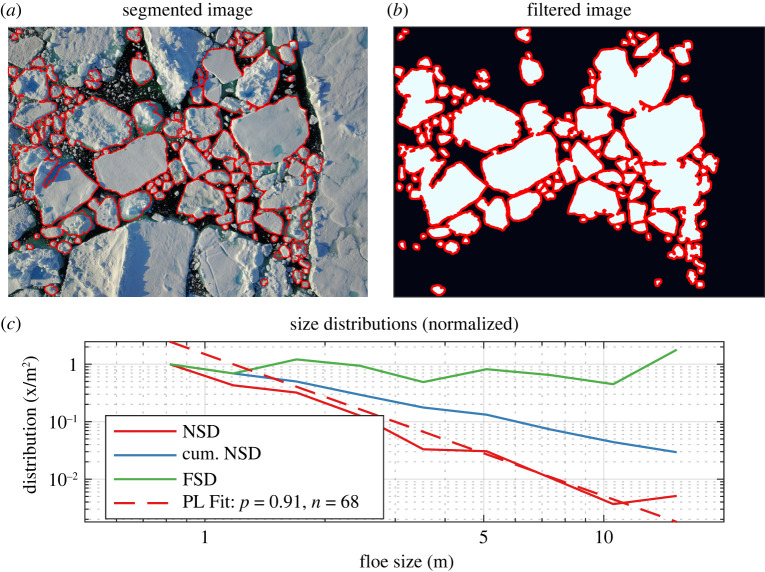
(Left) Floes identified using figure 1*a* from a dilation-filling-erosion procedure. (*a*) Original image, with floes identified outlined in red. (*b*) Binary, filtered image after erosion, filling, and dilation procedure applied. (*c*) Distributions (normalized to appear on the same axis) for identified floes. The power-law fit, and *p*-value (n=68) obtained via the algorithm of [39] is applied to the floe data and plotted as a dashed red line. A p-value above 0.05 means the hypothesis of a power-law distribution is plausible given the data. (Online version in colour.)

Generally, observations require a two-dimensional image to be pre-processed (to remove clouds or land, for example). The image is then analysed using a series of different filtering and processing steps to identify individual floes (e.g. [38]). Such a filtering method was applied to the floe field in figure 1*a*, which records floe sizes ranging from 0.75 m to 18 m (code to perform this segmentation is provided as electronic supplementary material). The image is first converted to black and white by means of a local brightness threshold, and individual connected components are identified. Along the boundary of each component, a small circular stencil is removed (the erosion step), which removes or disconnects some components. Components are then re-identified, and the same stencil is added at their boundaries (the dilation step) to preserve initial areas. The result of this procedure is shown as figure 2*a*,*b*. Floes intersecting the image boundaries are excluded. The FSD obtained from this procedure is given in figure 2*c*, as well as the number size and cumulative number distribution, normalized to 1. The number size distribution is fit to a power law using the POWERLAW [39] toolbox, and shown as a dashed red line.

Manual delineation of floes, such as done by Rothrock & Thorndike [28], can give more confidence in floe shapes and sizes, but this process is painstaking. Given the approximate scale invariance of the FSD, increasing observed resolution by a factor of 2 can increase the total number of floes by 10 or more. Despite this, some recent measurements have been made using supervised processing in this way [40].

### (b) From altimetry

An alternative to observing the FSD from two-dimensional imagery is satellite altimetry. Altimeters, like CRYOSAT-2 and ICESat-2, make straight-line measurements of the sea ice surface multiple times per day, emitting radar (as in CRYOSAT-2) or laser (as in ICESat-2) pulses. A key feature of both is their high precision in distinguishing sea ice from open water, although returns can be contaminated by clouds over the footprint of each return, which is approximately 300 m for CRYOSAT-2 and 10 m for ICESat-2 sea ice segments. Across the ice surface, altimeters can measure the length of continuous segments of sea ice [41]. Such ‘chord lengths’ can be related to the two-dimensionally derived FSD by an application of conditional probabilities. An early example of this methodology was given by Rothrock & Thorndike [28], and a version designed for radar altimetry was implemented and used with 8 years of CryoSAT-2 data in [41,42].

Altimetric reconstructions have the advantage of being completely unsupervised and that they make repeat passes in polar orbits. For example, ICESat-2 makes approximately 15 orbits per day, intersecting each cell in the 25 km grid explored in [42] approximately once per week. Yet the inversion of one-dimensional data to the FSD requires two critical assumptions: that the altimeter is passing over a region frequently enough to permit an FSD reconstruction, and that the altimeter is able to accurately delineate floe chords, subject to clouds that reduce the usable data. Challenges with floe chord delineation have been a point of focus using the ICESat-2 laser altimeter. While laser altimetry has extremely high along-track resolutions, work is ongoing to unify ICESat-2 and CryoSAT-2 observations [43], including comparisons with image-based reconstructions discussed above.

### (c) Observed floe size distributions in the Marginal ice zone

A great number of (at least four dozen) observational studies have characterized the FSD in different locations and times in polar seas, and exhaustive lists of such observations are given in reviews aimed at understanding commonalities in FSD shape in [31,37]. Fewer studies have looked specifically in the MIZ, where floes may be a metre or smaller across. This is a particular challenge in the Southern Ocean, where often the MIZ is formed of small pancake floes. As a result, observations that can accurately capture the required metre scales typically are ship-based (e.g. [26,44,45]) or helicopter-borne (e.g. [46]). These campaigns can be expensive and limited in their overall spatial and temporal coverage. For example, the dramatic evolution of the FSD captured in [47] was obtained in two helicopter flights spaced 4 weeks apart. Some studies blend multiple datasets (e.g. [36,48]) to increase the range of observed length scales, though these datasets do not always exactly overlap. Such is the challenge of this multi-scale problem.

In general, after floes are identified and catalogued, the FSD they form is fit to a power-law distribution across floe scales, under the assumption,

3.1 $$n(r)\propto r^{-\alpha },$$

over some size range. The question of the ‘power law hypothesis’ has been analysed in detail for sea ice measurements made away from the MIZ, and a great range of α values can and have been observed [30,31,37,49]. This review is not the place to energize the debate over the ‘power law hypothesis’. Still, an apparent consensus has emerged that power-law behaviour holds in regions affected by brittle fracture, such as in the Beaufort or Chukchi Seas [29,30,50,51]. It is clear that the FSD in the MIZ does not always have a power law tail, influenced as it is by small-spatial-scale processes that do not permit large floes—often the MIZ is comprised of a single peak floe size, or floes all near the same scale. Modelling and observational work aimed at reconstructing the FSD under the influence of waves has pointed to a lognormal or Gaussian distribution for the FSD in these regions [52–55].

### (d) Challenges and opportunities

Perhaps the main challenge in understanding FSD dynamics from observations is obtaining FSD evolution over a large spatial extent (enough to sample several decades of floe size) at high temporal resolution (sub-daily to daily) to isolate and examine individual FSD-evolving processes. This is particularly important in the MIZ, where floes are at their most dynamic and respond to episodic storm or swell events that may interfere with satellite-based imaging. Reconstructing the FSD from two-dimensional imagery is a painstaking, time- and data-intensive endeavour. Satellite altimetric data, which requires the compilation of multiple passes over a region, has its own issues with floe recognition and resolution, and to form an FSD requires multiple passes which might require long averaging windows. Airplane or helicopter flown altimetric or photographic data is limited by the ability to survey a wide region.

Recently, some new efforts to constrain floe size evolution have leveraged unique data locations or algorithms, such as floes broken by icebreaker-generated waves or in a protected estuary of the Gulf of St. Lawrence [56]. Relatively coarse-resolution MODIS imagery also allows for individual large floes (greater than 8 km) to be identified [57]. The latter can be used to study FSD evolution on daily time scales for such large floes, though has mainly been applied to examining upper-ocean vorticity expressed as floe motion.

## 4. Modelling the floe size distribution

Current sea-ice model design represents sea ice as an aggregate of many large floes [58], and therefore sea-ice models cannot represent the behaviour of individual floes. Thus to understand how sea ice floes evolve, sub-grid representations of sea ice geometry are required. FSD models evolve an equation,

4.1 $$\frac{Dn(r)}{Dt}=L_{T}(r)+L_{M}(r)+L_{W}(r),$$

where D/Dt is the material derivative following sea ice velocities, and $L_{T}$, $L_{M}$ and $L_{W}$ are floe size tendencies from thermodynamic effects, dynamic effects and wave effects, respectively. Recent efforts to understand and model FSD evolution have led to the generation of several FSD models, which can be broadly cast into three, non-mutually exclusive categories:

— Fixed models—assuming a standard shape of the FSD. Examples include [36,37], which relate FSD shapes to the solution to a simple mathematical equation, or [59], which relates the FSD shape to the seasonal evolution of sea ice concentration.
— Semi-diagnostic models—where key tendencies are fixed based on the evolution of other free-evolving variables. Examples include [34,60,61], where while advection and thermodnaymics are prognostically evolved, fractured sea ice is either uniformly distributed [60] or distributed as a power law [34,61].
— Prognostic models—where FSDs emerge from free-evolving tendency equations [32,62].

The semi-diagnostic and prognostic FSD schemes are implemented in global climate model codes that are used for both short-term and long-term climate study. These implementations, and some key features, are listed in table 1.

**Table 1.** A list of schemes for understanding FSD variability. ‘Redistribution’ refers to how the FSD is updated when a fracture event occurs. WWIII is the WAVEWATCH3 wave model [63].

model	type	redistribution	waves	implementation
PIOMAS-FSD [60,64]	semidiagnostic	uniform	parametric	PIOMAS [65]
WIPoFSD [34,66]	semidiagnostic	power-law	reanalysis w/[67]	NEMO-CICE
neXtSIM [61,68]	semidiagnostic	power-law	WWIII	neXtSIM [69]
HTR [32,33,62,70]	prognostic	super-parameterized	WWIII [63]	CESM2

### (a) Thermodynamics

A desired feature of all FSD models is their ability to represent the thermodynamic changes of sea ice related to floe size. Given a size-dependent lateral melt rate $\dot{r}(r)$, the thermodynamic evolution of sea ice floes under lateral melting can be determined exactly [32, see the derivation in §2.1],

4.2 $$L_{T}=-\frac{\partial }{\partial r}(\dot{r}n)+\frac{2\dot{r}}{r}n.$$

In practice, climate models simulate a single size-independent lateral melt rate (commonly called $w_{\text{lat}}$), one that is also constant across thickness categories [71]. The first term in equation (4.2) represents the ‘advection’ of floes between sizes as they melt laterally, and the second represents the impact of lateral melting on sea ice area. This latter term was first derived by Steele [25] in the case of all floes having the same size. Its impact is potentially large [71], but while it is implemented in sea ice models (CICE/LIM) that did not have an evolving FSD, the common assumption is that all floes were 300 m in radius or larger. This scale is too large for direct lateral melting to have an appreciable impact on sea ice evolution.

While lateral melting can significantly affect sea ice area and the shape of the FSD [72,73], the second term accounting for lateral melt’s impact on sea ice area is notably missing from [60]. In [34,61], the area effect is retained, but the requirement of fixed FSD shape means that lateral melting necessarily destroys large sea ice floes. Regardless, a crucial feature is retained by all: by simulating a multi-scale FSD, changes to that FSD resulting from fragmentation or wave-induced fracture impact sea ice thermodynamic evolution.

### (b) Fracture and redistribution

A second feature of FSD models is the representation of fracturing or fragmentation processes. The general form of a redistributive process is

4.3 $$L_{M}=-\int_{0}^{r}F(s;r)n(r)\;ds+\int_{r}^{\infty }F(r;s)n(s)\;ds,$$

where $F(r_{1};r_{2})$ describes the process of floes moving from size class $r_{1}$ to size class $r_{2}$. In general, the choice of F depends on the process being considered. For example, floe aggregation can be modelled using a three-partner coagulation equation [74], where $F(r,s)=K(r,s_{1},s_{2})$ as two floes of size $s_{1}$ and $s_{2}$ combine to make a floe of size r. This was derived for the case of floe riding and rafting by Horvat & Tziperman [32], and for the case of floe healing and pancake coagulation based on wave buoy data in [27].

These equations are most typically deployed to describe how sea ice breaks up through wave-induced fracture. This requires parameterization of a ‘participation function’ P(r), the likelihood that floes of size r will fracture, and a ‘redistribution function’ R(s;r), the resulting FSD formed from the fractured floe. Then F(s;r)=P(s)R(s;r). In the case of [60], for example, there is a uniform redistribution,

4.4 $$R(r;s)=\frac{1}{(c_{2}-c_{1})s}\;\text{for }c_{1}s\leq r\leq c_{2}s,$$

and zero otherwise, where $c_{1}$ and $c_{2}$ are empirically determined constants smaller than 1. The participation function P(r) is a cumulative distribution,

4.5 $$P(r)=1-\frac{1}{c_{b}}\int_{r}^{\infty }n(s)\;ds$$

or 0, whichever is greater, and $c_{b}$ is a ‘participation factor’ that varies with wind, ocean, and sea ice conditions (as the [60] model does not have an active wave component). The effect is a prioritization of participation by the largest FSD categories. For the case of wave-induced failure in coupled wave-ice models, [34,68] both adopt similar approaches: assuming a standard form of the FSD that can be parameterized using a few variables, generally a mean floe size and maximum floe size which evolve in response to the wave forcing. For example in [34], the FSD is a truncated power law with

4.6 $$N(r)\propto \left\{ \begin{array}{l} r^{-\alpha },\;d_{\text{min}}\leq r\leq l_{\text{var}}\\ 0\;\;\text{otherwise} \end{array}\right.$$

for a fixed α (set in [66] to be −2.56, matching Arctic FSD observations). The upper floe size limit $l_{\text{var}}$ is reduced to λ/2 for a peak wavelength λ, with waves fracturing sea ice following criteria outlined by Williams *et al*. [67].

A final approach is the prognostic HTR model, which employs a super-parameterized approach to wave-ice interactions (now released as a standalone model called WIFF [75]). Sea surface heights and sea-ice variability are explicitly generated in a one-dimensional configuration according to the local wave spectrum, and areas of high potential strain are computed to define a new FSD. This requires a number of iterations to converge as it is stochastic, making it expensive and challenging to use in a model global climate model. This was addressed through the use of a neural-net-based parameterization acceleration [75], which reproduces the effect of this module but at low computational cost. As examined in a companion paper in this issue [76], the WIFF model currently implemented in CICEv6 does not allow for the attenuation of waves within a grid cell and may erroneously lead to highly fragmented sea ice in the presence of locally generated wind waves. More work is needed to examine how such a fracture model can operate in this context.

### (c) Other processes

Other efforts have taken place to describe, for example, the healing of sea ice floes, their lateral *growth*, and the creation of new sea ice. For example, [27] developed a model of sea ice pancake growth and coagulation currently in use in the HTR FSD model from ship-borne imagery. A modification to the model of [33] in [66] parameterizes ‘brittle failure’ via an *ad hoc* restoring to a power-law distribution: sea ice area is transferred between neighbouring floe size categories at a specified rate if the local slope between them exceeds a scaling of $r^{-2}$.

## 5. Floe size distribution impacts

**Figure 3.**
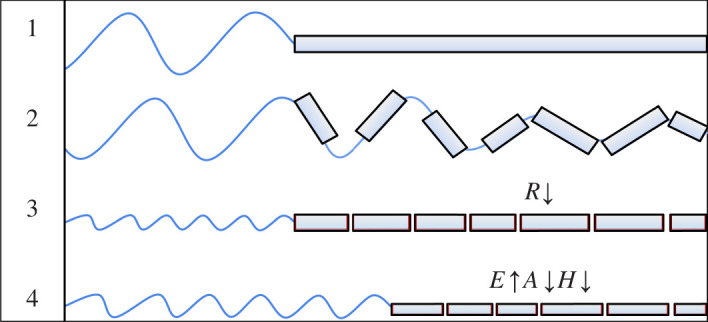
A summer floe-size-related positive feedback process in the MIZ. (1) A high-amplitude wave event reaches sea ice in the MIZ. (2) Sea ice flexes and fractures with the waves, (3) reducing its mean floe size R. This fractured sea ice is more susceptible to melting, reducing in area, A and thickness, H. It retreats, (4) potentially triggering a local albedo feedback, increased wave energies E, and the susceptibility of the sea ice to future wave events. (Online version in colour.)

A chief rationale for studying the FSD in coupled models and in observations is the possibility of climate-scale feedbacks triggered by FSD changes. One of the most-studied feedbacks is related to wave-induced fracture, summarized in figure 3 in the case of continuous, level ice

(1) An episodic wave event reaches the MIZ.
(2) Floes flexed by high-amplitude waves fail and are broken into smaller pieces.
(3) Sea ice becomes more susceptible to change, for example by
  (a) melting more rapidly [25,32,71],
  (b) consolidating more effectively [27,77],
  (c) being more mobile [78–81],
  (d) altering the attenuation and transmission of wave energy [22–24],
(4) The sea ice cover changes, triggering local coupled feedbacks, and:
  (P) Waves energize through increased fetch [82], reaching areas they previously could not, restarting the feedback process.
  (N) Changes to sea ice or wave properties allow for sea ice expansion, reducing wave energies and insulating a location against future wave events.

The positive side of this feedback process (4P) has been an area of research because outside of winter months sea ice in the MIZ is often in a state of melting. Thus waves can trigger local sea-ice-albedo feedbacks [72,83,84]. To date, studies have not considered whether the impact of waves might play a role, for example, in allowing sea ice expansion or strengthening sea ice against future wave events or loss. As smaller sea ice floes provide more surface to accrete frazil crystals and alter the transmission of wave energy [22,23], such a negative feedback may exist in winter months. Laboratory or ‘natural laboratory’ experiments (for example [56,85]) can help analyse these feedbacks and the role of the FSD in more controlled conditions.

### (a) Sea ice thermodynamics

Floe size variability can dramatically affect the way sea ice evolves as a thermodynamic medium, by preconditioning or increasing its susceptibility to melt, especially in concert with wave-derived fragmentation. For example, as explored in [72,84], a summer storm passing through a region fully covered by sea ice fractured it and led to a massive melt event spanning several days. Fracture by atmospheric processes is directly implicated in increasing floe perimeter, directly proportional to lateral melt rates [25]. In summer months, leads or other areas between floes can rapidly warm [86], and the direct melting of sea ice along lateral boundaries can contribute as much to the local change of sea ice volume as vertical processes.

Consider a highly simplified sea ice region with an area fraction C, thickness H and volume per unit area V=CH (units metre). Suppose, given a solar heat forcing S (units $W\;m^{-2}$) we parameterize the total heat available to melt sea ice in the ocean as proportional to sea ice area,

5.1 $$Q=S[C(1-\alpha_{\text{ic}})+(1-C)(1-\alpha_{\text{oc}})],$$

where $\alpha_{\text{ic}/\text{oc}}$ are the sea ice and ocean surface albedos, respectively. The time rate of change of sea ice volume per unit area is (time derivatives of X are given by $\dot{X}$)

5.2 $$\dot{V}=-\frac{Q}{\rho_{0}L_{f}}\equiv {\dot{V}}_{l}+{\dot{V}}_{b},$$

where ${\dot{V}}_{l}$ is the volume loss through lateral melting, ${\dot{V}}_{b}$ the volume lost through basal melting, with a sea ice density $\rho_{0}$ (units $\mathrm{kg}\;m^{3}$) and latent heat of freezing $L_{f}$ (units $J\;{\mathrm{kg}}^{-1}$). Define a relationship between lateral and basal fluxes, i.e.

5.3 $${\dot{V}}_{l}=\beta (P,H){\dot{V}}_{b},$$

where P is the sea ice perimeter density, and so heat is partitioned between lateral and basal melting according to some parameter β(P) that depends on the perimeter of sea ice β∈[0,∞). Noting $\dot{C}={\dot{V}}_{l}/H$, we obtain

5.4 $$\dot{Q}=-\frac{S\Delta \alpha }{H}{\dot{V}}_{l},$$

for $\Delta \alpha =\alpha_{\text{ic}}-\alpha_{\text{oc}}$. Taking the time derivative of equation (5.2) and noting that $\beta \dot{V}=(1+\beta ){\dot{V}}_{l}$ yields the coupled equations,

5.5 $$\ddot{V}\equiv \frac{H_{0}\dot{C}}{\tau }\;\mathrm{and}\;\dot{C}=\frac{\beta }{1+\beta }\frac{C\dot{V}}{V}.$$

Where we define a ‘fast’ melting timescale $\tau =S\Delta \alpha /\rho_{0}L_{f}V_{0}$ for an initial volume per square metre $V_{0}=H_{0}$, assuming all solar heat is used to melt sea ice. In the case β=0, the equations reduce to $\ddot{V}=0$. In that case, defining initial conditions $C(0)\equiv C_{0},Q(0)\equiv Q_{0},V(0)\equiv V_{0};\dot{V}(0)\equiv {\dot{V}}_{0}\equiv \rho_{0}L_{f}/Q_{0}V_{0}$, volume declines linearly as expected: $V(t)=V_{0}-{\dot{V}}_{0}t$ as all heat is used to melt the sea ice vertically.

A common definition of β is the ratio of basal surface area to lateral surface area [25], which can be expressed in terms of the number size distribution (assuming a fixed thickness $H_{0}$) [32] as

5.6 $$\beta =\frac{\int_{0}^{\infty }2\pi H_{0}rN(r)\;dr}{\int_{0}^{\infty }\pi r^{2}N(r)\;dr}=\frac{\int_{0}^{\infty }2H_{0}n(r)/r\;dr}{\int_{0}^{\infty }n(r)\;dr}\equiv 2H_{0}\frac{\left\langle r^{-1}\right\rangle }{c},$$

where ⟨⋅⟩ refers to an inner product with n(r). Assuming a δ function distribution of the FSD with all floes of size $r_{0}$ leads to $\beta =2H_{0}/cr_{0}$.

Equation (5.5) is integrated forward using initial conditions and parameter choices typical of sea ice in the summer Arctic: a fully ice-covered region of 2 m high sea ice under direct sunlight: $C_{0}=1,H_{0}=2\;m$, $S=320\;W\;m^{-2}$. We specify sea ice parameters using standard values, $L_{f}=3.36\times 10^{6}\;J\;{\mathrm{kg}}^{-1}$ and $\rho_{0}=910\;\mathrm{kg}\;m^{-3}$. We prescribe a sea ice albedo for snow-covered sea ice: $\alpha_{\text{ic}}=85\%$, for an initial absorbed heat flux of $Q_{0}=S(1-\alpha_{\text{ic}})=50\;W\;m^{-2}$, and prescribe an ocean albedo $\alpha_{oc}=6\%$. These choices yield a ‘fast’ melt timescale of τ≈23 d (if all heat is able to melt sea ice), and a ‘slow’ melt timescale $\tau^{*}=Q_{0}/\rho_{0}L_{f}V_{0}\approx 141\;d$ (if only vertical melting). The choice of parameters setting τ and $\tau^{*}$ bound the rate of sea ice loss as a function of floe size.

**Figure 4.**
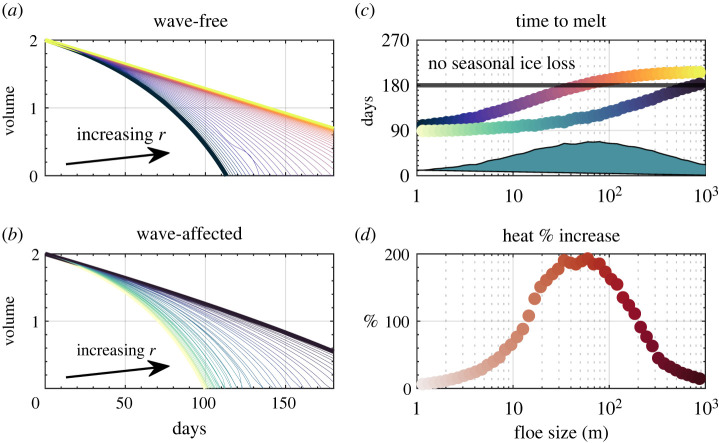
Output from a thermodynamic-wave feedback model. (*a*) Evolution of sea ice volume over time for different mean floe sizes, when floe sizes are fixed. (*b*) Same as (*a*), but the ensemble mean of stochastic wave-forced simulations where floe size evolves over time. (*c*) Time for sea ice to drop below $0.5\;m^{3}\;m^{-2}$ for both simulations as a function of the initial floe size. Colours in (*c*) correspond to lines in (*a*,*b*). Filled curve is the difference between the two scattered points. (*d*) Increase in upper-ocean heat uptake between ensemble of wave-affected and wave-free simulations, as a function of initial floe size. (Online version in colour.)

Figure 3*a* (black to yellow lines in direction of increasing initial floe size) shows curves of sea ice volume obtained by integrating forward equation (5.5). Reducing the floe size by three orders of magnitude greatly alters the response of overall sea ice. Ice melt is defined as the point where overall sea ice volume is less than $0.5\;m^{3}\;m^{-2}$. Figure 4*c* then scatters the day where that occurs as a function of floe size. The black line on (*c*) highlights a period of 180 days, which here approximately marks the duration of an entire melt season. Thus the difference between having small floes (as in the MIZ) or larger ones (as in the pack ice) can determine whether sea ice is melted over a seasonal cycle—from 1 m to 1 km, the time to melt sea ice is altered from 90 to 230 days. Integrations with large floes may sustain sea ice throughout the entire melt period. Such impacts have been observed in model studies: in simply varying floe sizes across an order of magnitude [71], in a model without active waves or the FSD, substantial changes were found in Arctic and Southern Ocean sea ice variability.

The ‘direct’ influence of lateral melting takes place for relatively small floes in the MIZ. Yet size-dependent effects are found for much larger scales, in the form of ‘apparent’ lateral melting via oceanic processes at the boundaries of floes hundreds of metres and larger. Because of on–off differences in solar forcing across a sea ice edge (often $100\;W\;m^{-2}$ and more), lateral gradients in temperature can develop in the ocean mixed layer, but not have an immediate effect on ocean circulation due to weak temperature control on density [87]. These gradients can build in time, becoming unstable to submesoscale baroclinic instabilities [88,89] that grow and mix heat laterally near the boundaries of sea ice floes. This small-scale variability has been observed in the MIZ [90], especially in the Southern Ocean via tagged seal data [91,92]. Eddy energization is directly proportional to the amount of perimeter of floes—as smaller floes are more readily influenced by eddies growing at their boundaries. Thus as floe sizes change over an order of magnitude, so too does the overall melt rate of sea ice. This is not necessarily due to direct lateral melting, but because of lateral advection of surface heat to the base of sea ice floes [89,93].

### (b) Wave propagation and impact

Floe size plays an important role in the propagation of ocean surface waves. Two main classes of approaches to wave-sea-ice impacts exist: those that treat sea ice as a viscous layer, and those that treat sea ice as a composite scatterer [16,94]. The viscous layer models are most appropriate when sea ice floe sizes are either much smaller than typical wavelengths, or much larger. However, an increasing number of theoretical, numerical, and observational studies evince that the impact of both wind-wave and swell energetics are to fracture sea ice floes into a lognormal distribution of floe sizes with modal peaks near the peak wavelength of the wave spectrum [52–56,73]. Thus waves may by their presence alter their own attenuation by changing the sea ice from an apparent viscous medium to an apparent scattering medium and back.

A number of different models have been employed to understand the way fractured floes affect wave attenuation. In general, these smaller floes attenuate less wave energy than larger ones, at both small and large wavelengths [22]. Thus the fracturing process by waves can enhance the propagation of waves into the MIZ, widening it and allowing for further propagation of waves in other storm events. This wave-only feedback process has not been studied *in situ*, but an examination of the seasonal variability of, for example, the MIZ width determined by floe sizes is a potentially motivating area for future work. Aspects of direct wave-sea-ice coupling are discussed in a number of articles in this issue [17,76,94].

### (c) Coupled wave-floe size distribution feedbacks

As discussed in figure 3, wave events in summer might alter the evolution of the sea ice cover in the MIZ by leading to enhanced melting. To explore this, consider the same model described in equation (5.5), but adding a stochastic ‘wave forcing’. For each individual integration, define a ‘wave impact’ time series W as an auto-regressive process of order 1 (a time-varying random process whose current value is affected by its previous value only, with a decorrelation timescale $\tau_{d}$), subjected to a wave shock ϵ,

5.7 $$W_{t}=\frac{1}{\tau_{d}}W_{t-1}+\theta (\epsilon -\epsilon_{c}).$$

The function ϵ is drawn from a uniform distribution from 0 to 1, θ is the heaviside function, and $\epsilon_{c}=1-\tau_{w}/\Delta T$ for the time step ΔT. Thus the probability of a ‘wave event’ that decays over a period $\tau_{d}$ is once per period $\tau_{w}$, and the effect of these waves decays over a timescale $\tau_{d}$. To realize the impact on sea ice melting, floe size evolution is parameterized as

5.8 $$\frac{\partial r}{\partial t}=\frac{1}{\tau_{H}}(r-r_{0})+\frac{W}{\tau_{b}}(r_{0}-r_{f}),$$

with a ‘healing’ timescale $\tau_{H}$, ‘breaking’ timescale $\tau_{b}$, fractured floe size $r_{f}$ and initial floe size $r_{0}$. The time-evolving r value is used to update β, and the forcing terms in equation (5.5). Each initial floe size is simulated with an ensemble of 30 members. We choose a typical storm return period of $\tau_{w}=14\;\mathrm{days}$ with a typical duration $\tau_{d}=1\;\mathrm{days}$ to mirror repeated impacts with cyclones in the Arctic. We choose a breaking timescale $\tau_{b}=6\;h$ to represent fast fracture processes in the presence of waves. We arbitrarily choose a healing timescale $\tau_{H}=7\;\mathrm{days}$, similar to the healing timescale used in [68]. The fractured floe size is set to 1 m, which is roughly the lower limit of floe size categories used in climate models. These parameter choices are chosen to examine the potential importance of this feedback, however some, for example the size of fractured floes, may be larger or vary in time. The choice of these parameters is only intended to be illustrative here, and the spread of outcomes can be sensitive to their choice. For example, figure 4 is reproduced using a fractured floe size of 10 m in the Supporting Data, with qualitative similarity to results shown below. All other model parameters are the same as in the wave-free case.

For each initial floe size, the ensemble-average results are plotted as blue-black curves in figure 4*b*—the impact of waves is a significant increase in the lateral melt rate of the sea ice in total. The same melt-period diagnostic based on these wave simulations is added in figure 4*c*, which shows that across the range of initial floe sizes, there is a significant decline in the period of sea ice melting. The solid curve plots the difference between the wave-free and ensemble-mean wave-influenced time to melt sea ice, which can be as high as 70 days for floes with an average size around 100 m. Figure 4*d* plots the relative increase in heat (Q(waves on)/Q(waves off)) absorbed by the ocean over 180 d, compared to simulations with the same initial floe size but without a wave forcing, equal to zero if there is no impact of waves fracturing the sea ice. For FSDs with floes ranging from 20 to 200 m, this impact can lead to a doubling or more of heat uptake in the MIZ.

The inclusion of wave effects can substantially alter regional feedbacks. For example, [62] showed that inclusion of coupled wave-FSD feedbacks results in alterations of sea ice thickness and concentration in both hemispheres of 5–25%, varying seasonally. Bateson *et *al*.* [34] found little change in seasonally integrated overall sea ice melting, but significant changes in the partitioning of melting between vertical and lateral processes as floe sizes in the MIZ became small. In assessments of the potential importance of thermodynamic feedbacks alone, [71] found the sensitivity to lateral melting to be key in altering the extent and thickness of sea ice in both hemispheres, primarily because it allows for open water formation—the mechanism explored in §5a. Examining the impact of sea ice fragmentation on rheology and wave radiation stress on sea ice motion [17,68] found high local dependence of sea ice coverage on changes to the FSD, but limited global effects. All analyses with active FSD components, however, used reanalysis atmospheric forcings that may limit feedbacks by determining the thermodynamic position of the sea ice edge. Long coupled wave-ice simulations, in particular those with an active atmosphere, have only started to be performed to assess climate-scale feedbacks. With improved FSD observations, and improved observations of waves in sea ice, mainly from the ICESat-2 altimeter [13,17], along with this new class of coupled models, we may begin to understand the FSD and its role in MIZ and polar climate processes more deeply.

### (d) Other feedbacks: ice dynamics, light and ecological change

Sea ice is a two-dimensional granular material. This means that the floe size plays the role of the grain size in standard granular theory [95]. A series of sea ice rheologies, for example [96] or [69], have been built to incorporate information about floe size (or an equivalent measure). The fracture of sea ice by waves may then influence the drift of sea ice in the MIZ. Sea ice floe sizes also play an important role in the transmission of momentum to the upper ocean, and the effect of floe sizes on ocean drag has been included in parameterizations of form drag [66,97,98]. Efforts are underway to understand how the FSD influences momentum transfer, sea ice rheology, and subsequent sea ice evolution in MIZs, which may have a pronounced impact on overall sea ice coverage. For example: in [17], sea ice drift can be substantially altered in the aftermath of energetic wave events. In [99], FSD-influenced granular mechanics are shown to play an important role in setting the width and variability of the MIZ.

More highly broken floes permit higher contact between the ice and the ocean, by the simple fact that they move independently, revealing small patches of open water. These small regions reduce total sea ice concentration and permit more sunlight. More floe-like systems, for example, in the Southern Ocean, may permit significant amounts of sunlight to reach through them in summer. This in turn could seed phytoplankton blooms underneath sea ice [100]. Indeed, fractured, thick sea ice was found to support phytoplankton blooms [101] in May–June, months before seasonal sea ice retreat, and lateral variability in light conditions plays an important role in driving the availability of sunlight in the upper ocean [6,102,103]. In the Southern Ocean, the floe-like mosaic that extends across the sea ice zone has a lower concentration than the Arctic, which may permit phytoplankton growth under sea ice throughout the spring and summer that is challenging to observe remotely [75,104].

## 6. Conclusion

Leveraging a litany of recent efforts to understand how floes influence sea ice and its role in the Earth system, many areas exist for further research into MIZs and the floes that comprise them. There remain limited high-temporal-resolution observations of FSD evolution, particularly when impacted by waves or under stress. Climate models are now only beginning to be able to evolve coupled wave-ice feedbacks, and remain far from able to resolve eddy processes influenced by and driving differential sea ice motion at the scale of floes. The potential impact of both positive and negative feedbacks related to sea ice floe evolution remain an under-explored topic with potentially high relevance as more of the Arctic becomes seasonally ice-free. There is much to be excited about as this field continues to grow and evolve.

## Data Availability

Code to produce figures in this manuscript is available at https://github.com/chhorvat/MIZ-Review-Figures. Electronic supplementary material is available online [105].

## Footnotes

1 Published, in English, and to this author’s knowledge.
